# Hip Preservation Surgery in Patients With Femoroacetabular Impingement Syndrome and Acetabular Dysplasia Improves Functional Measures and Pain Catastrophizing

**DOI:** 10.7759/cureus.52461

**Published:** 2024-01-17

**Authors:** John M Gaddis, Rafael de Souza, Benjamin Montanez, Paul A Nakonezny, Bretton Laboret, Ryan Bialaszewski, Joel E Wells

**Affiliations:** 1 Department of Orthopedic Surgery, University of Texas Rio Grande Valley School of Medicine, Edinburg, USA; 2 Department of Orthopedic Surgery, University of Texas Southwestern Medical Center, Dallas, USA; 3 Department of Population and Data Clinical Sciences, University of Texas Southwestern Medical Center, Dallas, USA; 4 Department of Orthopedic Surgery, Baylor Scott and White Health, McKinney, USA

**Keywords:** hip preservation surgery, acetabular dysplasia, femoroacetabular impingement syndrome, hip function outcomes, pain catastrophization

## Abstract

Background

Chronic hip pain is a debilitating condition that severely reduces one’s quality of life. Prior studies uncovered a link between hip pathologies and pain catastrophizing, anxiety, and depression. The purpose of this study was to investigate whether hip preservation surgery in patients with femoroacetabular impingement syndrome (FAIS) and acetabular dysplasia (AD) improves functional outcomes and pain catastrophizing.

Methods

Patients with FAIS and AD were requested to complete a hip questionnaire both preoperatively and postoperatively at a single academic center (University of Texas Southwestern Medical Center, Dallas, Texas, USA). Pain catastrophizing was evaluated using the pain catastrophizing scale, and pain level was assessed using the visual analog scale. Assessments of hip functional outcomes included the hip outcome score (HOS) and the hip disability and osteoarthritis outcome score (HOOS). Outcome measures before and after treatment were compared using the dependent samples t-test. A correlation analysis, using the Spearman partial correlation coefficient (rs), was conducted to evaluate the relationship between variables.

Results

The results indicated a clinically significant improvement in functional measures and pain catastrophizing in patients who underwent hip preservation surgery. The most significant discovery was an inverse relationship between both HOOS quality of life (rs=-0.293, p=0.0065, false discovery rate (FDR)=0.0210) and HOS activities of daily living (rs=-0.242, p=0.0254, FDR=0.0423) and pain catastrophizing; however, similar improvements were seen in pain catastrophizing with improvements in other functional outcomes.

Conclusion

Undergoing hip preservation surgery for patients with AD or FAIS improved their hip functional measures and decreased pain catastrophizing postoperatively. The improvement of hip function, quality of life, and pain catastrophizing reveals an intricate link between the functional outcomes of hip preservation surgery and pain catastrophizing.

## Introduction

According to National Health Interview Survey data conducted in 2016, 20.4 million US adults are affected by chronic pain [1]. Chronic pain is one of the most common reasons adults seek medical care and is closely associated with the development of psychological disorders such as anxiety and depression [1]. Hip pathologies span a wide range of demographics and are a major cause of debilitating chronic pain, leading to loss of function and productivity [2]. Prior studies display a link between hip pathologies and higher levels of pain catastrophizing, anxiety, and depression [3,4]. Pain catastrophizing has previously been defined as a heightened threat value of the pain stimulus, feeling helpless in the setting of pain, and the incapability of controlling pain-related thoughts before, during, or after a painful event [5]. In addition, higher levels of depression, anxiety, and pain catastrophizing have been correlated with worse surgical outcomes, increased postoperative opiate use, and longer hospital stays [6-8].

Femoroacetabular impingement syndrome (FAIS) and acetabular dysplasia (AD) are two common hip pathologies predominantly affecting younger demographics, with sequelae including osteoarthritis and chronic hip pain [9]. After exhausting non-surgical conservative measures, patients often undergo hip preservation procedures such as hip arthroscopy, surgical hip dislocation, and periacetabular osteotomy. The primary objective of these procedures is to preserve function, prevent the development of arthritic damage, and prolong the lifespan of the hip joint [10]. Studies have shown that hip preservation procedures lead to improvements in pain and functional scores in patients with actively symptomatic hips [9,11,12]. Studies have also revealed that lower functional scores correlate with higher pain catastrophizing and depression at the time of a clinical diagnosis [4,11-13]. Furthermore, Gudmundsson et al. previously determined that patients with various hip pathologies exhibit improvements in pain catastrophizing with increases in patient-reported hip function [14]. However, to our knowledge, there are no prior studies looking at how postoperative function impacts pain catastrophizing scores in patients with AD and FAIS undergoing hip preservation surgery, specifically.

Therefore, the goal of this study was to investigate hip function both pre- and postoperatively and determine if there was a relationship between improved functional scores and pain catastrophizing in patients who underwent hip preservation surgery for FAIS or AD. We hypothesized that patients with improved postoperative functional scores after surgery would similarly benefit from reduced pain catastrophizing.

This article was previously posted to the Research Square preprint server on September 14, 2023.

## Materials and methods

We retrospectively evaluated all patients undergoing hip preservation surgery at a single academic medical center (University of Texas Southwestern Medical Center, Dallas, Texas, United States) between January 2016 and January 2020. Following institutional review board approval, all patients provided written consent and underwent assessments using validated measurement scales pre- and postoperatively. The psychometric assessment utilized was the pain catastrophizing scale (PCS) [15]. The functional assessments included the hip outcome score (HOS), the hip disability and osteoarthritis outcome score (HOOS), and the visual analog scale (VAS) [16-18].

The University of Texas Southwestern Medical Center Institutional Review Board approved the study (approval number: STU122016-058). Once consented, 2,493 total IRB-approved, electronic, self-report hip questionnaires were sent out to patients who underwent hip preservation surgery. There were 1,487 responses from unique patients with a diagnosis of AD or FAIS for which they received hip preservation surgery at our center. Of these, 1,399 patients were excluded on the basis of missing a pre- or postoperative survey or being incomplete. From the original sample, we conducted a complete enumeration, including all 88 patients diagnosed with AD or FAIS in our study. No patients refused to participate in the study or elected to drop out. All patients in the study presented with hip pain and were diagnosed based on clinical and radiological examination by the treating physician and senior author (JW), an orthopedic surgeon specializing in hip preservation and reconstruction. AD was diagnosed on radiographic evidence of femoral head uncovering, a lateral center-edge of Wiberg angle <20°, and patients presenting with symptomatic AD. The exclusion criteria consisted of those with an additional and/or different diagnosis than AD. FAIS was diagnosed based on clinical symptoms, a physical exam associated with FAIS, or imaging findings such as pincer or cam deformities and/or an alpha angle >55° [9]. Exclusion criteria included those with an additional and/or different diagnosis than FAIS or patients with physical exams or radiographic findings not associated with FAIS. All patients were assessed pre- and postoperatively after isolated hip preservation surgeries, including hip arthroscopy, surgical hip dislocation, and periacetabular osteotomy. All patients who underwent periacetabular osteotomy, hip arthroscopy, or surgical hip dislocation for AD or FAIS from January 2016 to January 2020 were eligible for inclusion. Additional exclusion criteria included patients with prior trauma.

The primary outcomes collected were pain catastrophizing and patient-reported hip functionality in the pre- and postoperative periods. Pain catastrophizing was evaluated using the PCS. The PCS consists of 13 items and asks participants to evaluate past painful experiences and indicate the degree to which they experienced certain thoughts and feelings on a scale from zero to four. The scale assesses rumination, magnification, and helplessness. The total score can be calculated by summing the responses to the 13 items, and the scores range from zero to 52. A PCS score of ≥30 is indicative of clinically relevant pain that is catastrophizing [15]. The patient's subjective pain level was assessed using the VAS for pain. The VAS allows patients to subjectively rate current pain level, typical/average pain, and pain level at its best and at its worst on a scale from zero to 10 (zero = “no pain at all,” 10 = “pain as bad as it can be”) [19].

Patient-reported levels of function were quantified using the HOS and the HOOS scores. The HOS is broken up into activities of daily living (HOS ADL) and sports (HOS sports) subscales. Respectively, each subscale has 17 and nine items that are scored from zero to four (zero being unable to perform; four being no difficulty). Subscale total scores are summed and normalized so that the final scores are a percent of the maximal function [20]. The HOOS consists of 40 questions separated into six subscales related to pain (HOOS pain), other symptoms (HOOS symptoms), function in ADLs (HOOS ADL), function in sports and recreation (HOOS sports), and hip-related quality of life (HOOS QOL) [21]. For our study, each item was scored from zero to four (zero indicating extreme trouble and four meaning no trouble), and the sum was normalized so that a score of zero indicates severe impairment and a score of 100 represents no problems. Additional patient-level variables obtained from the patient's electronic medical record (EMR) included sex, age, and body mass index (BMI, kg/m^2^). The American Society of Anesthesiologists (ASA) classification of physical status was assessed using patient comorbidities listed in the EMR.

An initial pool of 18 characteristic variables was selected for analysis as potential predictors of pain catastrophizing in patients with AD and FAIS. The pool of potential covariates included age, sex, BMI, Tonnis grade, ASA grade, history of surgery on current hip, diagnosis, comorbidities, PCS total, PCS rumination, PCS magnification, PCS helplessness, VAS pain, HOS ADL, HOOS symptoms, HOOS stiffness, HOOS sport, and HOOS QOL.

Statistical analysis

Demographic and clinical characteristics for the sample of 88 patients with a range of hip pathologies were described using the sample mean and standard deviation for continuous variables and the frequency and percentage for categorical variables. The mean level of pain catastrophizing and functional outcome measures at pretreatment and posttreatment were compared using the dependent samples t-test. Next, a correlation analysis using the Spearman partial correlation coefficient (rs) was conducted to evaluate the relationship between change in self-reported pain catastrophizing and change in patient-reported functional outcome measures while controlling for age, BMI, and time in days from pre- to post-treatment assessment. Statistical analyses were carried out using SAS software version 9.4 (SAS Institute, Inc., Cary, NC). The level of significance was set at α=0.05 (two-tailed), and we implemented the false discovery rate (FDR) procedure, where applicable, to control false positives over multiple tests [22].

## Results

The sample of 88 patients was 80.68% (n=71) female, with a mean age of 32.44±13.36 years (range: 13-72 years). The mean BMI was 26.30±4.96 kg/m^2^. The sample was made up of 35 patients with AD (39.77%) and 53 with FAIS (60.23%). Of the 88 patients, 32 (36.36%) had previous surgery on the current hip. Low back pain was the most prevalent comorbidity (n=44, 50.00%), while 15 (17.05%) patients had depression. The demographic and clinical characteristics of the 88 patients in the current study are shown in Table 1.

**Table 1:** Demographic and clinical characteristics of the overall sample The data has been represented as N: number of patients, %: percentage of N, M: mean, and SD: standard deviation. ASA: American Society of Anesthesiologists classification, AD: acetabular dysplasia, BMI: body mass index

Characteristic	Overall sample (n=88)
Patient demographics	
Age, years, M (SD)	32.44 (13.36)
Female gender, % (n)	80.68% (71)
Patient factors	
BMI, kg/m^2^, M (SD)	26.30 (4.96)
Tonnis grade, % (n)	(n=16)
0	62.50% (10)
1	37.50% (6)
2	0% (0)
3	0% (0)
ASA classification, % (n)	(n=10)
1	100% (10)
2	0% (0)
3	0% (0)
4	0% (0)
History of surgery on current hip, % (n)	36.36% (32)
Diagnosis groups	(n=88)
Femoroacetabular impingement, % (n)	60.23% (53)
AD, % (n)	39.77%(35)
Patient comorbidities, % (n)	(n=88)
Low back pain	50% (44)
Osteoporosis	3.41% (3)
Fractures	4.55% (4)
Depression	17.05% (15)
High blood pressure	7.95% (7)
Cancer	3.41% (3)
Anemia	3.41% (3)
Lung disease	1.14% (1)
Heart disease	0%(0)
Liver disease	0%(0)
Kidney disease	0% (0)
Diabetes	1.14% (1)
Ulcer/stomach disease	6.82% (6)

The dependent sample t-test revealed a significant change or improvement in mean levels of pain catastrophizing and functional outcomes from pre- to post-treatment for patients with adverse hip conditions. The HOOS Stiffness, HOOS Sport, and HOOS QoL showed the greatest improvements in the patient assessments. The HOOS stiffness, HOOS sport, and HOOS QoL had increased mean changes in outcomes of 16.47, 13.28, and 16.67, respectively. The VAS pain scale had a preoperative mean of 5.30 and a postoperative mean of 2.95, which showed an average decrease of 2.35 on the pain scale. For the PCS total, the preoperative mean and postoperative mean were 18.27 and 10.22, respectively, showing a decrease of 8.04. The mean HOS ADL increased from 63.52 preoperatively to 74.55 postoperatively. These results are shown in Table 2.

**Table 2:** Change in mean levels of pain catastrophizing and function from pre- to post-treatment for patients with adverse hip conditions N: number of patients, M: sample mean, SD: standard deviation, ΔM: mean change in outcome, PCS: pain catastrophizing scale, VAS: visual analog scale, HOS: hip outcome score, HOOS: hip disability and osteoarthritis outcome score, ADL: activities of daily living, QoL: quality of life. Change was operationally defined as post-minus pre-level. p-value (two-tailed): dependent samples t-test was used to test for differences in sample means from pre- to post-treatment. FDR values were all 0.0001. A p-value is considered significant at p<0.05

Outcome	N	Pretreatment	Post-treatment	Δ_M_	
		M (SD)	M (SD)	M(SD)	p-value
PCS total	88	18.27 (12.62)	10.22 (10.80)	-8.04 (11.55)	<0.0001
PCS rumination	88	6.42 (4.71)	3.84 (3.98)	-2.58 (4.50)	<0.0001
PCS magnification	88	3.41 (2.79)	2.00 (2.56)	-1.41 (2.73)	<0.0001
PCS helplessness	88	8.01 (5.70)	4.38 (5.06)	-3.62 (5.34)	<0.0001
VAS pain	88	5.30 (2.15)	2.95 (2.24)	-2.35 (2.71)	<0.0001
HOS ADL	88	63.52 (18.13)	74.55 (20.54)	11.02 (23.31)	<0.0001
HOOS symptoms	88	42.33 (19.19)	46.16 (17.65)	3.83 (27.17)	<0.0001
HOOS stiffness	88	30.39 (21.17)	46.87 (23.23)	16.47 (27.76)	<0.0001
HOOS sport	88	27.58 (18.69)	40.86 (19.14)	13.28 (27.74)	<0.0001
HOOS QoL	88	15.72 (14.01)	32.38 (18.74)	16.67 (23.35)	<0.0001

The Spearman partial correlation coefficients revealed a significant inverse relationship between change in level of function (as measured by the HOOS QoL) and change in pain catastrophizing total (rs=-0.293, p=0.0065, FDR=0.0210), pain catastrophizing rumination (rs=-0.391, p=0.0002, FDR=0.0010), pain catastrophizing magnification (rs=-0.225, p=0.0380, FDR=0.1900), and pain catastrophizing helplessness (rs=-0.339, p=0.0015, FDR=0.0075), while controlling for age, BMI, and time in days from pre- to post-treatment assessment. Pain catastrophizing improved as the level of function (QoL) improved. Spearman correlation coefficients also revealed that pain catastrophizing improved with improvement in the other patient-reported functional outcomes (Table 3). However, of the functional outcomes, the Spearman correlation coefficients revealed that improvement in HOOS QoL along with HOOS stiffness and HOS ADL can be interpreted as having a greater magnitude of relative importance in the expected relationship with improvement in pain catastrophizing, including rumination, magnification, and helplessness.

**Table 3:** Spearman correlation coefficients (rs) between the change in PCS with the change in functional outcomes Change was operationally defined as post-minus pre-level. p-value: two-tailed test on Spearman rho. FDR: false discovery rate, N: number of patients, rs: Spearman rho, PCS: pain catastrophizing scale, HOOS: hip disability and osteoarthritis outcome score, HOS: hip outcome score, ADL: activities of daily living, QoL: quality of life. p-value is considered significant at p<0.05

Change in level of function	N	r_s_	p-value	FDR	N	r_s_	p-value	FDR	N	r_s_	p-value	FDR	N	r_s_	p-value	FDR
	Change in PCS total				Change in PCS rumination				Change in PCS magnification				Change in PCS helplessness			
HOOS QoL	88	-0.293	0.0065	0.0210	88	-0.391	0.0002	0.0010	88	-0.225	0.0380	0.1900	88	-0.339	0.0015	0.0075
HOOS symptoms	88	-0.095	0.3858	0.3897	88	-0.236	0.0293	0.0366	88	-0.051	0.6384	0.6384	88	-0.112	0.3058	0.3058
HOOS stiffness	88	-0.284	0.0084	0.2010	88	-0.315	0.0033	0.0083	88	-0.184	0.0904	0.2260	88	-0.306	0.0043	0.0108
HOOS sport	88	-0.094	0.3897	0.3897	88	-0.100	0.3622	0.3622	88	-0.110	0.3127	0.4318	88	-0.197	0.0699	0.0874
HOS ADL	88	-0.242	0.0254	0.0423	88	-0.284	0.0084	0.0140	88	-0.103	0.3454	0.4318	88	-0.273	0.0115	0.0192

## Discussion

Multiple studies reveal the link between chronic pain and mental disorders such as anxiety and depression [4,5,23]. Psychological symptoms may impact patients’ perceptions of their disabilities, pain, and treatment outcomes across a wide range of orthopedic pathologies [4-6,23,24]. In patients with hip pathologies, the literature reveals connections between lower reported function and higher levels of anxiety, depression, and pain catastrophizing [4,11-14]. Specifically, in the FAI population, pre-existing mental health disorders have been associated with worse outcomes in patients undergoing hip arthroscopy. These patients were found to have altered pain reduction and a reduced chance of returning to active military service [25].

The aim of this study was to fill the current gap in knowledge regarding whether orthopedic surgical interventions directed toward improving function also improve pain catastrophizing. Studies exploring other orthopedic pathologies, such as adolescent idiopathic scoliosis, displayed improvement in psychometric scores following correction [26], whereas studies investigating shoulder surgery displayed persistent psychological disorders postoperatively in a majority of patients [27]. Given the mixed results, it is important to study each orthopedic disorder individually in relation to pain catastrophizing. This enables providers to tailor patient expectations based on their specific pathology and operation. Specifically, we investigated patients with FAI and AD undergoing hip preservation procedures. Acknowledging the close link between mental health disorders and chronic pain may enable orthopedic providers, pain management specialists, physical therapists, and mental health providers to approach various pathologies from a multidisciplinary perspective, resulting in better outcomes.

Previous studies have indicated that hip preservation surgeries result in clinically significant improvements in function and pain measures [28]. Our study confirmed this finding and found significant improvements, particularly in HOS ADL, HOOS symptoms, HOOS stiffness, HOOS sport, HOOS QoL, and VAS pain scale. Furthermore, the results of this study align with the results of previous studies indicating that preoperative patients with FAIS and AD display clinically significant levels of pain catastrophizing [4]. Importantly, pain catastrophizing levels improved across all three scales (rumination, magnification, and helplessness) and significantly decreased postoperatively in patients with FAIS and AD undergoing hip preservation surgery.

The most revealing aspect of this study was the link established between improvements in HOOS QoL and HOS ADL and pain catastrophizing. ADL includes basic functions such as getting into and out of a car or going up a flight of stairs. These are activities that are essential to the independence of an individual. Additionally, these activities are performed frequently; thus, in patients with hip pathologies such as FAIS and AD, limitations in performing these activities serve as regular reminders of their disability. The physical burden of a hip pathology affects the patient’s quality of life and serves as a detriment to their psychological health, worsening the measures of rumination, magnification, and helplessness.

The results of this study reveal an intricate link between the functional outcomes of hip preservation surgery and pain catastrophizing. This further emphasizes the necessity to approach both physical and psychological pathologies from a multidisciplinary approach. Studies investigating hip preservation surgeries have shown that patient expectations of a surgical procedure vary according to patient demographics and the clinical characteristics of their pathology. Specifically, younger age, shorter symptom duration, and worse hip-functional status were linked to higher expectations [29]. Studies have also shown that recovery expectations in other musculoskeletal pathologies are highly associated with future work participation and recovery outcomes [30]. Thus, the importance of establishing clear patient expectations for a surgical procedure cannot be underestimated. It is the responsibility of the healthcare provider to present patients with adequate information regarding expected improvements in not only functional but also psychological outcomes. Further studies are needed to investigate other unique hip pathologies and their treatments across various modalities (physical therapy, medical, and surgical) in association with functional and psychometric outcomes.

This study has limitations. Only one academic center was utilized in this study, and the patients recruited presented to only one specialist hip clinic. Therefore, the results of this study may not be generalizable to all FAIS or AD patients. Second, the results of this study are susceptible to selection bias. From a cohort of 1,487 AD or FAIS patients, 1,399 were excluded. These patients were excluded primarily because they were lost to follow-up or due to inadequately completed surveys. Furthermore, the hip questionnaire given to participants did not evaluate any pharmacologic therapy patients may be receiving for depression or anxiety, and this may have interfered with the results.

## Conclusions

In this study, patients living with AD or FAIS were found to have poor hip function scores and high levels of pain catastrophizing, confirming prior studies. Undergoing hip preservation surgery for patients with AD or FAIS improved their hip function and decreased their overall pain level. Furthermore, hip preservation surgery improved pain catastrophizing levels on all three scales assessed. Most significantly, a correlation was found between increased hip functional measures and decreased pain catastrophizing postoperatively. This indicates that when patients experienced improved quality of life and were able to complete ADL with more ease, they felt more able to be in control of their pain level and were less likely to feel helpless in managing their symptoms.

Future studies can investigate potentially confounding variables, including psychosocial factors such as depression, employment, social support structure, and income, in relation to functional improvements following orthopedic procedures. Future studies can also compare psychometric improvements in orthopedic pathologies across various treatment modalities such as pharmacologic, physical therapy, and surgical interventions. Finally, different levels of pain catastrophizing can be explored in patients undergoing primary hip surgery versus patients undergoing a second surgery on the same hip. Pain catastrophizing can play a substantial role in how patients perceive their surgical success. Improving hip function through hip preservation surgery has been shown to significantly decrease patient levels of pain catastrophizing postoperatively in AD and FAIS populations, further underscoring the benefit of surgical hip preservation treatments.
